# Hair Silicon as a Long-Term Mineral Exposure Marker in Coronary Artery Disease: A Pilot Study

**DOI:** 10.3390/nu17243956

**Published:** 2025-12-18

**Authors:** Ewelina A. Dziedzic, Łukasz Dudek, Andrzej Osiecki, Jakub S. Gąsior, Wacław Kochman

**Affiliations:** 1 Cardiovascular Clinic, Centre of Postgraduate Medical Education, 01-813 Warsaw, Poland; 2 Department of Cardiology, Bielański Hospital, 01-809 Warsaw, Poland; 3 Department of Pediatric Cardiology and General Pediatrics, Medical University of Warsaw, 02-091 Warsaw, Poland

**Keywords:** silicon, trace minerals, hair analysis, coronary artery disease, cardiometabolic risk, subclinical inflammation, oxidative stress

## Abstract

**Background**: Coronary artery disease (CAD) is a multifactorial atherosclerotic disorder. Silicon (Si) is a trace mineral with potential antioxidant, anti-inflammatory, and lipid-modulating effects, but its clinical relevance in cardiovascular disease remains unclear. This study evaluated whether hair Si concentration—reflecting long-term exposure—is associated with CAD severity, clinical phenotype, risk factors, and systemic inflammation. **Methods**: A total of 130 patients with angiographically confirmed CAD (N = 36, 28% women) who met the inclusion criteria were enrolled. Disease severity was quantified using the Coronary Artery Surgery Study Score (CASSS) and SYNTAX score. Hair Si concentration was determined by inductively coupled plasma optical emission spectrometry (ICP-OES). Associations with demographic, clinical, biochemical, and inflammatory parameters were analyzed using non-parametric tests and multivariable ordinal logistic regression. **Results**: Median hair Si concentration was 21.3 ppm (range: 0.7–211.0). Hair Si levels showed no significant differences across CAD severity assessed by CASSS (H = 2.51; *p* = 0.47) or SYNTAX score (r = 0.079; *p* = 0.37). Similarly, no differences were observed between patients with stable angina and those presenting with acute coronary syndrome (*p* = 0.57) or between individuals with and without prior myocardial infarction. Hair Si concentration was unrelated to age, BMI, cardiovascular risk factors, lipid profile, or systemic inflammatory indices (all *p* > 0.2). **Conclusions**: Hair silicon concentration was not associated with CAD severity, phenotype, or systemic inflammation, suggesting that long-term Si exposure is metabolically neutral in advanced atherosclerosis. Unlike other minerals, silicon appears unlikely to serve as a diagnostic or prognostic biomarker in CAD, although its relevance may be confined to early vascular remodeling and primary prevention.

## 1. Introduction

Coronary artery disease (CAD) remains the leading cause of cardiovascular mortality worldwide [1,2,3]. It is a multifactorial atherosclerotic disorder characterized by endothelial dysfunction, lipid accumulation, oxidative stress, and chronic inflammation [4,5,6]. Beyond classical risk factors, increasing evidence highlights the importance of nutritional and metabolic factors in modulating cardiovascular risk. The balance of essential and trace elements plays a pivotal role in endothelial function, oxidative balance, and vascular remodeling [7,8,9].

Silicon (Si) is a dietary trace element that plays both a structural and potentially regulatory role in connective tissue metabolism and vascular homeostasis [10]. Present mainly as orthosilicic acid, with serum levels reflecting recent intake rather than long-term exposure [11,12], Si is involved in the synthesis and cross-linking of glycosaminoglycans, proteoglycans, and collagen fibers [13,14,15], contributing to extracellular matrix (ECM) stability and vascular integrity essential for maintaining vascular elasticity and endothelial function.

Dietary silicon intake is derived primarily from whole grains, certain vegetables, and beverages rich in soluble silica, including mineral waters and beer [16]. Its bioavailability depends on chemical form and gastrointestinal conditions such as pH, solubility, and intestinal transit time [17,18]. Urinary silicon excretion correlates positively with dietary intake, confirming efficient renal elimination [19]. Aging and chronic cardiometabolic conditions can reduce silicon absorption and increase urinary excretion, resulting in lower systemic availability [19,20]. Biomarkers reflecting long-term nutritional exposure, such as hair mineral analysis, may provide more accurate estimates of habitual Si status than serum measurements, especially in older individuals [20].

Experimental data suggest Si deficiency may promote atherosclerotic changes by weakening ECM stability, enhancing lipid deposition, and disturbing proteoglycan architecture [21,22,23,24,25]. In animal studies, decreased aortic Si content correlated with lipid accumulation and elastic fiber degeneration [22,23,26,27]. Molecularly, Si modulates inflammatory and oxidative processes by downregulating TNF-α, iNOS, and COX-2 expression, suppressing NF-κB activation, and normalizing antioxidant enzyme activity [28,29,30], suggesting adequate Si intake may help preserve vascular structure and reduce pro-inflammatory signaling in early atherogenesis. However, excessive exposure to crystalline silica (SiO_2_) exerts endothelial toxicity and pro-oxidant effects, illustrating a dose-dependent influence of silicon on vascular health [31,32,33].

Most available evidence derives from experimental studies, whereas human data remain limited and largely observational [30,34,35,36]. In a randomized, placebo-controlled trial involving healthy elderly individuals, silicon-enriched Spirulina supplementation significantly improved arterial elasticity and reduced pulse wave velocity in participants with elevated systolic blood pressure [36]. Si has also been shown in animal models to beneficially modulate lipid metabolism and blood pressure [22,24,27], and to improve glucose tolerance [10,23,25,37]. Notably, serum Si levels decline with age and exhibit sex-related differences [38], while hair concentration displays marked interindividual variability [39].

Hair mineral analysis provides noninvasive assessment of long-term trace element status, reflecting cumulative exposure rather than short-term dietary fluctuations [40,41], and is increasingly applied in cardiovascular and metabolic research [42]. However, the relationship between long-term silicon status—reflected by hair Si concentration—and CAD severity or systemic inflammation remains unexplored.

This pilot study aimed to evaluate the relationship between hair silicon concentration and CAD severity, assessed by the Coronary Artery Surgery Study Score (CASSS) and SYNTAX score, as well as its associations with cardiovascular risk factors and systemic inflammatory indexes (NLR, MLR, PLR, SIRI, SII). We hypothesized that higher hair Si levels would be associated with lower CAD severity and reduced systemic inflammation (H_1_), whereas the null hypothesis assumed no significant relationship (H_0_). Clarifying this relationship could enhance our understanding of nutritional modulation of coronary atherosclerosis and support future dietary or supplementation strategies targeting cardiometabolic health.

## 2. Materials and Methods

### 2.1. Study Population

This observational, cross-sectional study included a total of 130 consecutive patients (36 women and 94 men) who: met the inclusion criteria, underwent diagnostic coronary angiography at the Department of Cardiology, Bielański Hospital, Warsaw, Poland, between 2013 and 2017 and have angiographically confirmed CAD. All participants were residents of Warsaw and reported no occupational exposure to silicon.

The study was conducted in accordance with the Declaration of Helsinki and approved by the Bioethical Committee of the Medical University of Warsaw (KB/124/2014). All participants provided written informed consent. The authors declare no external funding and no conflicts of interest.

### 2.2. Exclusion Criteria

Patients were excluded if the proximal 3-cm hair segment (measured from the scalp) had been dyed, chemically treated, or subjected to procedures such as permanent waving. Chemical modification of the hair shaft is known to interfere with ICP-OES measurements. Importantly, hair length and natural structure (straight, wavy, or curly) were not exclusion criteria; only chemically altered hair was excluded. Other exclusion criteria included active cancer, acute viral or bacterial infections, chronic kidney disease (stages III–V), elevated inflammatory markers (CRP > 10 mg/L), autoimmune or inflammatory diseases, terminal illness, history of thrombosis or restenosis, and supplementation with silicon or other trace elements. Participants who used shampoos containing mineral additives were also excluded to avoid potential contamination bias.

### 2.3. Laboratory Tests and Clinical Data

Venous blood samples were processed and analyzed within two hours of collection. Laboratory tests included complete blood count, fasting glucose concentration, and serum lipid levels (total cholesterol [TC], low-density lipoprotein cholesterol [LDL], high-density lipoprotein cholesterol [HDL], and triglycerides [TG]).

The diagnosis of type 2 diabetes mellitus or prediabetes followed the 2019 ESC Guidelines on diabetes, prediabetes, and cardiovascular disease [43]. Hyperlipidemia was defined according to the 2019 ESC/EAS Guidelines for the management of dyslipidemias [44]. Hypertension was diagnosed in accordance with the 2024 European Society of Hypertension clinical practice guidelines [45].

Nutritional status was assessed using body mass index (BMI), calculated as weight in kilograms divided by height squared in meters (kg/m^2^). Obesity was defined as BMI ≥ 30 kg/m^2^ [46].

### 2.4. Inflammatory Indices

Inflammatory indices were calculated using complete blood count parameters obtained on admission, according to previously established formulas and validated publications. The following indices were assessed:

- NLR = neutrophil-to-lymphocyte ratio [47],
- MLR = monocyte-to-lymphocyte ratio [48],
- PLR = platelet-to-lymphocyte ratio [49],
- SII = (platelet × neutrophil)/lymphocyte [50],
- SIRI = (neutrophil × monocyte)/lymphocyte [51].

### 2.5. Hair Sample Analysis

Hair samples (~0.2–0.3 g) were collected from the occipital region, cut close to the scalp, and consisted of undyed hair. Samples were washed using a non-ionic detergent (Triton X-100, Sigma Aldrich Sp. z.o.o., Poznań, Poland) water solution (1:100) in an ultrasonic bath for 5 min, rinsed with high-purity water, acetone, and water, and then dried to constant mass. Dry samples (0.15 g) were digested with 4 mL of 65% nitric acid (Merck, Darmstadt, Germany) and 1 mL of 30% hydrogen peroxide (Merck) in closed polypropylene tubes (8 mL) and incubated at 80 °C for 30 min in a microwave digestion system (MarsXpress, CEM Corp., Stallings, NC, USA). After cooling, samples were diluted to a final volume of 10 mL with Milli-Q water and analyzed using an inductively coupled plasma optical emission spectrometer (ICP-OES; iCAP7400, Thermo Fisher Scientific, Waltham, MA, USA). Certified standards (CGZN1 and CGCU1, Inorganic Ventures, Christiansburg, VA, USA) were used for calibration and quality control. The analytical precision and accuracy were verified using blank and triplicate samples. Results were expressed as µg/g of dry weight. The sample preparation and analytical protocol followed the methodology previously described by our group in the study of hair calcium levels in patients with coronary artery disease [52].

### 2.6. Coronary Angiography

All participants underwent coronary angiography via either the radial or femoral access route to assess the extent of coronary artery stenosis. Based on angiographic findings, patients were referred for revascularization when clinically indicated, with percutaneous coronary intervention (PCI) being the preferred revascularization strategy [53]. The diagnosis of acute coronary syndrome (ACS) was established according to the 2020 ESC Guidelines [54]. ACS was diagnosed when there was an elevation in cardiac troponin above the 99th percentile of the upper reference limit, together with one or more of the following: symptoms of myocardial ischemia, new ischemic ECG changes, development of pathological Q waves, imaging evidence of myocardial necrosis, or angiographic detection of an intracoronary thrombus. The severity of coronary atherosclerosis was quantified using both the SYNTAX score and the Coronary Artery Surgery Study Score (CASSS). The SYNTAX score considers lesion complexity, spatial distribution, and hemodynamic relevance [55,56]. The CASSS assigns 1 point for ≥70% stenosis in any major coronary artery (LAD, LCx, RCA) and 2 points for ≥50% narrowing of the left main coronary artery, classifying disease into single-, double-, or triple-vessel CAD [57].

### 2.7. Statistical Analysis

Normality of data distribution was evaluated using the Shapiro–Wilk test. Depending on distribution, differences between groups were assessed using Pearson’s chi-square or Fisher’s exact test for categorical variables, and the Mann–Whitney U test or Kruskal–Wallis test with Dunn’s post hoc correction for continuous variables. Correlations between silicon concentration and clinical parameters were analyzed using Spearman’s rank correlation coefficient (R). Statistical significance was defined as two-tailed *p* < 0.05. Analyses were conducted using Statistica 13.3 (StatSoft Inc., Tulsa, OK, USA).

## 3. Results

Details on the study group were presented elsewhere recently [52]. The median Si concentration was: 21.3 parts per million (ppm) (range: 0.7–211.0).

**Figure 1:**
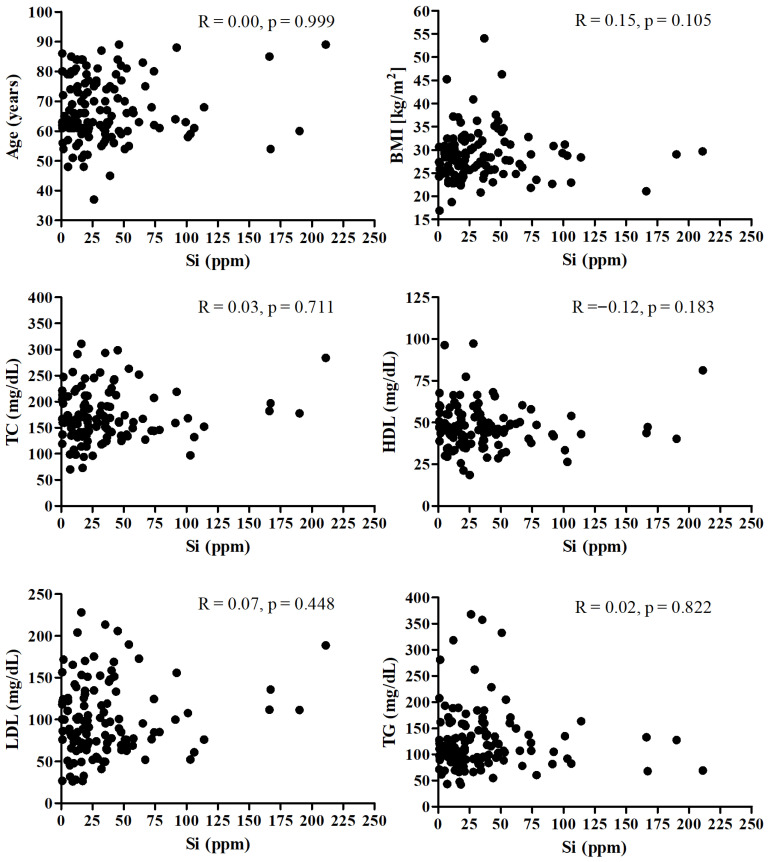
Correlation between the Si and age, BMI, and lipid profile.

Figure 1 presents the correlation between the Si and age, BMI, and lipid profile. There was no significant correlation between SI and mentioned parameters.

**Figure 2:**
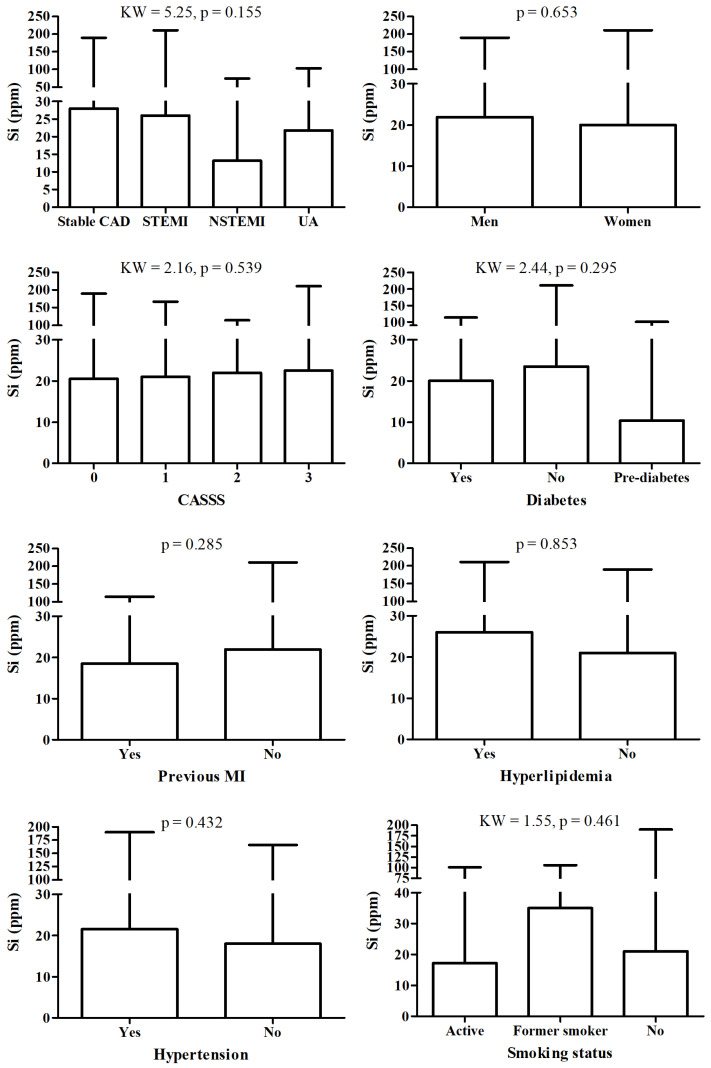
Si level according to different diagnosis and/or clinical status.

There were no significant changes in Si between patient according to detailed diagnosis, sex, CASS score, diabetes, hyperlipidemia and hypertension status, nor according to smoking status and fact of MI in history (Figure 2).

**Figure 3:**
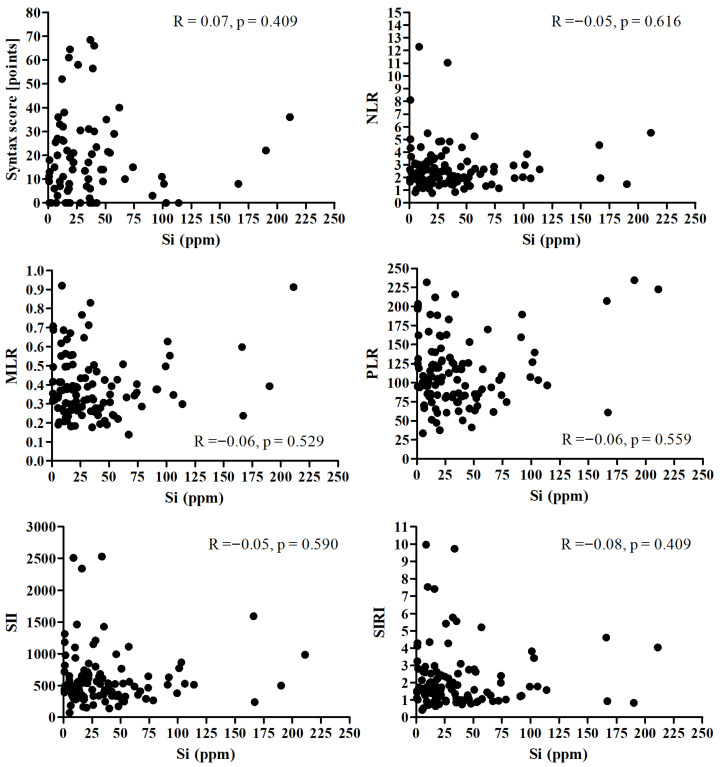
Correlation between the Si and Syntax score and all analyzed inflammatory markers.

There was no significant correlation between Si and Syntax score and all analyzed inflammatory markers (Figure 3).

## 4. Discussion

### 4.1. Principal Findings

In this pilot study, hair silicon (Si) concentration—a marker of long-term nutritional and environmental mineral exposure—showed no significant association with the presence, clinical phenotype, or angiographic severity of coronary artery disease (CAD). Hair Si levels did not differ between patients with stable angina and those with acute coronary syndromes (STEMI, NSTEMI, or unstable angina) and were not correlated with the CASSS or SYNTAX score, which quantify atherosclerotic lesions extent and complexity.

Furthermore, Si concentration was independent of traditional cardiometabolic risk factors, including age, body mass index, hypertension, diabetes or prediabetes, dyslipidemia, and smoking status, as well as of lipid profile and systemic inflammatory indexes (NLR, MLR, PLR, SIRI, SII). These results indicate that long-term silicon status appears metabolically inert in patients with established CAD, and does not reflect disease severity, vascular inflammation, or metabolic background.

To our knowledge, this is the first study to assess the relationship between hair silicon levels and both angiographic and clinical features of CAD in a well-characterized cardiometabolic cohort, providing a novel nutritional perspective on trace element homeostasis in coronary atherosclerosis.

### 4.2. Comparison with Previous Studies

Our findings are consistent with earlier data showing no clear link between tissue or hair silicon levels and atherosclerotic burden. Kwaśny et al. [39] reported lower mean hair Si concentration in atherosclerotic patients than in healthy controls, but wide interindividual variability and overlapping ranges (10–20 µg/g) limited its diagnostic usefulness. Similarly, recent analyses in angiographically confirmed CAD demonstrated significant associations of calcium, magnesium, or nickel—but not silicon—with lesion extent or localization [58,59]. Comparable results were reported in peripheral arterial disease [60].

Epidemiological studies on occupational exposure to crystalline silica (SiO_2_) have yielded inconsistent cardiovascular outcomes. In a large Swedish cohort, long-term exposure to respirable crystalline silica was associated with a higher risk of acute myocardial infarction in women (HR = 1.66; 95% CI 1.27–2.18), but not in men [61]. Other reviews confirmed that excessive SiO_2_ exposure can trigger macrophage activation, NLRP3 inflammasome signaling, oxidative stress, and endothelial dysfunction, potentially enhancing cardiovascular risk [33]. Conversely, Esfahani et al. [62] found no clear association between silica exposure and cardiovascular mortality.

These studies addressed toxicological rather than physiological exposures, suggesting a biphasic effect: under normal conditions, Si may support ECM stability and vascular elasticity, whereas excessive SiO_2_ exposure exerts pro-inflammatory and pro-atherogenic actions. This aligns with the growing recognition in nutritional cardiology that the biological impact of trace elements follows a U-shaped dose–response relationship, with both deficiency and overload being detrimental.

### 4.3. Biological Mechanisms and Potential Role of Silicon

Silicon functions as a structural trace mineral, contributing to collagen–proteoglycan network stabilization and ECM integrity [63]. These effects are especially relevant during early vascular remodeling, when Si supports elastin and collagen organization and preserves arterial elasticity. Experimental evidence indicates that silicon attenuates lipid peroxidation and oxidative stress, suppresses NF-κB-dependent inflammation, and reduces the expression of TNF-α, COX-2, and iNOS in vascular tissues [28,30], potentially contributing to vascular homeostasis and endothelial protection under physiological conditions. However, in advanced atherosclerosis, dominated by chronic inflammation, lipid deposition, and calcification, the structural and antioxidant role of Si becomes marginal. Recent review [36] and histochemical analyses confirm that aortic Si content declines with plaque progression but does not mirror inflammatory activity [26]. Moreover, limited intestinal absorption and rapid renal excretion constrain Si bioavailability [16]. Consequently, hair Si concentration reflects cumulative nutritional exposure rather than metabolically active tissue pools. These findings suggest silicon may exert a structural and potentially protective role during early vascular remodeling, but its contribution to advanced CAD appears minimal. From a nutritional standpoint, maintaining adequate dietary Si intake may be more relevant for vascular resilience and endothelial integrity during the early, preclinical atherosclerosis stages.

### 4.4. Associations Between Hair Silicon and Cardiometabolic Risk Factors

#### 4.4.1. Lipid Profile

In this CAD cohort, most individuals presented dyslipidemia, while only a minority exhibited a normal lipid profile. No significant associations were found between hair silicon (Si) content and serum lipid parameters, suggesting that Si does not reflect lipid-related metabolic status nor participate in compensatory mechanisms of dyslipidemia in advanced CAD. Experimental studies demonstrate that silicon supplementation may improve lipid metabolism in animals by reducing atherosclerotic plaque formation and lipid peroxidation [64], lowering LDL and triglycerides, and increasing HDL concentrations [24,27]. However, these effects remain unconfirmed in humans, limited to experimental or preclinical models. These findings indicates Si is nutritionally neutral in established dyslipidemia; yet, adequate dietary Si intake may play a role in lipid regulation during earlier, pre-atherosclerotic disease phases. The lack of interventional data evaluating silicon supplementation in lipid disorders underscores our study’s novelty, as one of the first clinical evaluations of Si–lipid relationships in established CAD.

#### 4.4.2. Glucose Metabolism

Despite high diabetes and prediabetes prevalence, no significant association was observed between hair Si content and glucose metabolism disturbances, suggesting previously reported antidiabetic effects of Si in animal models [34] are not replicated in clinical populations with advanced cardiometabolic disease. Experimental data indicate that Si may improve glucose tolerance, enhance insulin sensitivity, and modulate adipokine signaling, but these effects may be masked by antidiabetic pharmacotherapy and the complex metabolic milieu characteristic of CAD. Future studies incorporating treatment type and glycemic control markers (e.g., HbA1c) could clarify metabolic interactions between Si and glucose homeostasis. From a nutritional standpoint, bioavailable Si from whole grains, cereals, and mineral waters may support glucose–insulin balance in earlier disease stages, warranting investigation in primary prevention settings.

#### 4.4.3. Blood Pressure and Vascular Function

Hypertension was highly prevalent (86%), yet no relationship between hair Si content and blood pressure was identified, indicating that Si does not reflect hemodynamic status nor antihypertensive therapy effectiveness in CAD. Although animal studies show Si supplementation can reduce systolic blood pressure and influence vascular remodeling–related gene expression [23], these benefits remain unconfirmed in humans. A recent randomized trial demonstrated that Si-enriched Spirulina improved arterial stiffness in healthy elderly individuals [36], suggesting Si may enhance vascular elasticity under physiological conditions. The absence of such effects in our cohort suggests a window of nutritional responsiveness limited to earlier disease stages, before irreversible atherosclerotic remodeling predominates.

#### 4.4.4. Body Mass and Energy Metabolism

Despite high overweight and obesity prevalence, Si levels did not correlate with BMI, supporting the notion that body mass does not determine hair Si status in advanced CAD. Experimental studies propose indirect metabolic links between Si and insulin resistance via proteoglycan-mediated pathways [22], but such mechanisms remain undemonstrated in humans. Thus, Si appears nutritionally and metabolically inert regarding anthropometric determinants of cardiometabolic risk.

#### 4.4.5. Sex-Related Differences

No sex-related differences in hair Si content were observed (*p* = 0.598). Prior studies suggest hormonal modulation of silicon metabolism, with lower Si reported in postmenopausal women and variable patterns in osteoarthritic disease [65]. Given our cohort’s advanced age, hormonal influences may be attenuated, reducing sex-dependent variability. Potential interactions between estrogen status and Si bioavailability deserve evaluation in younger and postmenopausal populations.

#### 4.4.6. Age

Age showed no correlation with hair Si content (R = −0.027; *p* = 0.757), consistent with prior reports [39]. Although tissue and serum Si decline with age [10,38], hair analysis reflects long-term cumulative exposure rather than short-term metabolic turnover, explaining the discrepancy. These results support utility of hair mineral profiling as a biomarker of habitual rather than acute nutritional status.

#### 4.4.7. Smoking and Oxidative Stress

No association was identified between smoking and hair Si content, indicating that Si is not a responsive biomarker of oxidative or environmental stress in CAD [35]. Unlike zinc or selenium, Si does not participate in antioxidant enzyme systems, potentially explaining its metabolic inertia under oxidative stress.

### 4.5. Silicon and Subclinical Inflammation

No significant associations were observed between hair Si concentration and subclinical inflammatory indices (NLR, MLR, PLR, SII, SIRI), recognized surrogate markers of systemic inflammation [66,67,68]. This suggests long-term Si status does not reflect vascular inflammation intensity in established CAD, particularly noteworthy given that chronic immune activation constitutes a central atherogenesis mechanism [69]. Experimental and preclinical data demonstrate that bioavailable silicon may modulate immune and redox pathways, including attenuating reactive oxygen species generation, suppressing COX-2, TNF-α, and iNOS expression, and inhibiting NF-κB activation [28,30,70]. Moreover, high dietary Si intake has been associated with altered monocyte activity and reduced pro-inflammatory cytokine release [71,72]. In rheumatoid arthritis patients, higher Si status is linked to reduced oxidative stress and lower IL-6 levels [35]. However, the absence of comparable associations in our CAD cohort suggests potential anti-inflammatory actions of Si may be restricted to the early atherogenesis stages or other immune-mediated disorders. Advanced atherosclerosis—characterized by sustained inflammation, lipid-rich plaques, and vascular calcifications—likely represents a stage where silicon homeostasis modulation exerts minimal influence on systemic or vascular inflammatory activity. Furthermore, hair mineral analysis primarily reflects long-term environmental and nutritional exposure rather than the metabolically active Si pool within vascular tissues, potentially explaining the neutral associations observed. These results indicate that silicon does not participate in active immunometabolic processes typical of advanced coronary atherosclerosis, supporting its primarily structural rather than regulatory role in this context.

### 4.6. Summary and Interpretation

Despite high prevalence of cardiometabolic abnormalities—including hypertension, dyslipidemia, obesity, and diabetes—none influenced hair Si content, indicating Si remains independent of metabolic and inflammatory determinants of advanced atherosclerosis. Although Si appears nutritionally neutral in established CAD, experimental and early-stage clinical evidence suggests potential roles in vascular elasticity, ECM integrity, lipid metabolism, and glucose homeostasis prior to overt disease. Si may retain preventive potential in early endothelial dysfunction or metabolic syndrome, warranting longitudinal nutritional studies. Future prospective studies integrating dietary Si intake assessment, bioavailability from different food sources, and interventional trials are needed to determine whether optimal silicon exposure supports vascular resilience and cardiometabolic prevention in earlier disease stages.

### 4.7. Limitations and Future Directions

This study has several limitations. Its cross-sectional and single-center design precludes causal inference and does not allow assessment of dynamic changes in silicon metabolism. Hair mineral analysis primarily reflects long-term nutritional and environmental exposure rather than metabolically active silicon pools. The study cohort consisted mainly of older individuals with advanced coronary artery disease, in whom potential early vascular effects of Si may no longer be detectable. Pharmacotherapy, particularly with statins, antidiabetic, and antihypertensive agents, may also influence trace element homeostasis. Moreover, the relatively small sample size, typical for pilot studies, may limit generalizability.

An additional limitation concerns the temporal structure of the dataset. Although patient recruitment occurred between 2013 and 2017, the present analysis was completed later due to an expanded research protocol, which included incorporation of SYNTAX/CASSS scoring, addition of inflammation-based indices, and reprocessing of angiographic data. Earlier publications from this cohort addressed different research questions and did not include these components [39]. These factors should be taken into account when interpreting the temporal context of the study.

Future research should integrate dietary intake assessment, serum and tissue Si quantification, and validated nutritional questionnaires to better define the relationship between silicon status and cardiovascular health. Longitudinal and interventional studies are warranted to determine whether dietary silicon or supplementation can influence early vascular remodeling, oxidative stress, or endothelial function. An integrative approach combining biochemical, clinical, and nutritional data will help determine whether silicon plays a protective or merely structural role in cardiometabolic homeostasis.

## 5. Conclusions

In this pilot study, hair silicon concentration—a marker of long-term nutritional and environmental exposure—showed no association with the severity or clinical phenotype of coronary artery disease (CAD). Silicon levels were independent of traditional cardiometabolic risk factors, lipid profile, and systemic inflammatory indexes, suggesting a metabolically neutral role in patients with established atherosclerosis. Although experimental evidence indicates that silicon may exert structural, antioxidant, and anti-inflammatory effects during early vascular remodeling, such actions appear to be of limited relevance in advanced CAD. Future longitudinal and interventional studies integrating dietary intake assessment, serum silicon quantification, and nutritional profiling are warranted to clarify whether silicon contributes to vascular protection or metabolic homeostasis in the earlier stages of cardiometabolic disease and whether its adequate intake may hold preventive relevance.

## Data Availability

The original contributions presented in the study are included in the article, further inquiries can be directed to the corresponding author.
